# Genome Sequence of *Legionella massiliensis*, Isolated from a Cooling Tower Water Sample

**DOI:** 10.1128/genomeA.01068-14

**Published:** 2014-10-16

**Authors:** Isabelle Pagnier, Olivier Croce, Catherine Robert, Didier Raoult, Bernard La Scola

**Affiliations:** Unité de Recherche sur les Maladies Infectieuses et Tropicales Emergentes, CNRS, UM63, CNRS7278, INSERM U1095, IRD 198, Faculté de Médecine, Aix-Marseille Université, Marseille, France

## Abstract

We present the draft genome sequence of *Legionella massiliensis* strain LegA^T^, recovered from a cooling tower water sample, using an amoebal coculture procedure. The strain described here is composed of 4,387,007 bp, with a G+C content of 41.19%, and its genome has 3,767 protein-coding genes and 60 predicted RNA genes.

## GENOME ANNOUNCEMENT

*Legionella massiliensis* strain LegA^T^ was isolated from an environmental water sample from a cooling tower located in South France, using an amoebal coculture procedure (1). This is a Gram-negative and Gimenez-positive bacillus classified in the genus *Legionella*. Based on the sequencing of the complete 16S rRNA gene, using BLASTn (2), the closest related species is *Legionella birminghamensis* (accession no. NR044953), with 96.7% similarity. Regarding the macrophage infectivity potentiator (*mip*) gene, strain LegA^T^ exhibits 78% similarity with *Legionella feeleii* (accession no. FJ009368). The strain *L. massiliensis* LegA^T^ is unable to grow on Columbia with 5% sheep blood agar and is able to grow after 3 days on buffered charcoal yeast extract (BCYE) medium under a 5% CO_2_ atmosphere. The bacteria showed negative reactions for oxidase, cefinase, and gelatinase. *L. massiliensis* LegA^T^ is deposited in the DSMZ (DSM 24804^T^) and CSUR (SCUR P146^T^) culture collections.

We therefore sequenced the whole genome of *L. massiliensis* LegA^T^ in order to determine the phylogenetic relationships with closely related *Legionella* species. The DNA genome was sequenced using two high-throughput next-generation sequencing (NGS) technologies: Roche 454 (3) and MiSeq Illumina (Illumina, Inc., San Diego, CA). A library of 5-kb paired-ends was constructed, loaded on a PicoTiterPlate (PTP), and sequenced with the Roche-GS FLX Titanium sequencing kit XLR70. MiSeq Illumina sequencing was performed using two applications, paired-end and mate-pair Nextera libraries, in a 2 × 250 bp run for each barcoded library.

The reads from various sequencing technologies were first assembled separately. The reads from 454 sequencing were assembled into contigs and scaffolds using Newbler version 2.8 (Roche, 454 Life Sciences). The Illumina reads were trimmed using Trimmomatic (4) and then assembled with the SPAdes software (5, 6) while adding contigs generated from Roche 454. The obtained contigs were combined by the SSPACE (7) and Opera softwares (8) and helped by GapFiller (9) to reduce the set. Some manual refinements using CLC Genomics software (CLC bio, Aarhus, Denmark) and homemade tools improved the genome. Finally, the draft genome of *L. massiliensis* LegA^T^ consists of 8 contigs without gaps, containing 4,387,007 bp and a G+C content of 41.19%.

Noncoding genes and miscellaneous features were predicted using RNAmmer (10), ARAGORN (11), Rfam (12), Pfam (13), and Infernal (14). Coding DNA sequences (CDSs) were predicted using Prodigal (15), and functional annotation was achieved using BLAST+ (16) and HMMER3 (17) against the UniProtKB database (18). The genome was shown to contain at least 60 predicted RNAs, including 7 rRNAs, 40 tRNAs, 1 transfer-messenger RNA (tmRNA), and 12 miscellaneous RNAs. A total of 3,767 genes were also identified, representing a coding capacity of 3,811,533 bp (coding percentage, 86.88%). Among these genes, 174 (4.62%) were founded as putative proteins and 1,326 (35.2%) were assigned as hypothetical proteins. Moreover, 2,405 genes matched a least one sequence in the Clusters of Orthologous Groups (COGs) database (19, 20) with BLASTp default parameters.

### Nucleotide sequence accession numbers.

The *L. massiliensis* strain LegA^T^ genome sequence has been deposited at EMBL under the accession numbers CCVW01000001 to CCVW01000008.
